# A New Anaesthetic Protocol for Adult Zebrafish (*Danio rerio*): Propofol Combined with Lidocaine

**DOI:** 10.1371/journal.pone.0147747

**Published:** 2016-01-25

**Authors:** Ana M. Valentim, Luís M. Félix, Leonor Carvalho, Enoque Diniz, Luís M. Antunes

**Affiliations:** 1 Laboratory Animal Science Group, Instituto de Biologia Molecular e Celular (IBMC), Universidade do Porto, Porto, Portugal; 2 Instituto de Investigação e Inovação em Saúde, Universidade do Porto, Porto, Portugal; 3 Centro de Investigação e Tecnologias Agroambientais e Biológicas (CITAB), Universidade de Trás-os-Montes e Alto Douro, Vila Real, Portugal; 4 Departamento de Sanidade Animal (DSA), Faculdade e Medicina Veterinária (FMV), Universidade José Eduardo dos Santos (UJES), Huambo, Angola; National University of Singapore, SINGAPORE

## Abstract

**Background:**

The increasing use of zebrafish model has not been accompanied by the evolution of proper anaesthesia for this species in research. The most used anaesthetic in fishes, MS222, may induce aversion, reduction of heart rate, and consequently high mortality, especially during long exposures. Therefore, we aim to explore new anaesthetic protocols to be used in zebrafish by studying the quality of anaesthesia and recovery induced by different concentrations of propofol alone and in combination with different concentrations of lidocaine.

**Material and Methods:**

In experiment A, eighty-three AB zebrafish were randomly assigned to 7 different groups: control, 2.5 (2.5P), 5 (5P) or 7.5 μg/ml (7.5P) of propofol; and 2.5 μg/ml of propofol combined with 50, (P/50L), 100 (P/100L) or 150 μg/ml (P/150L) of lidocaine. Zebrafish were placed in an anaesthetic water bath and time to lose the equilibrium, reflex to touch, reflex to a tail pinch, and respiratory rate were measured. Time to gain equilibrium was also assessed in a clean tank. Five and 24 hours after anaesthesia recovery, zebrafish were evaluated concerning activity and reactivity. Afterwards, in a second phase of experiments (experiment B), the best protocol of the experiment A was compared with a new group of 8 fishes treated with 100 mg/L of MS222 (100M).

**Results:**

In experiment A, only different concentrations of propofol/lidocaine combination induced full anaesthesia in all animals. Thus only these groups were compared with a standard dose of MS222 in experiment B. Propofol/lidocaine induced a quicker loss of equilibrium, and loss of response to light and painful stimuli compared with MS222. However zebrafish treated with MS222 recovered quickly than the ones treated with propofol/lidocaine.

**Conclusion:**

In conclusion, propofol/lidocaine combination and MS222 have advantages in different situations. MS222 is ideal for minor procedures when a quick recovery is important, while propofol/lidocaine is best to induce a quick and complete anaesthesia.

## Introduction

In the last decade the number of publications in zebrafish tripled, from 847 in 2004 to 2509 in 2014 (Pubmed database; keyword: “zebrafish” in Title/Abstract), being the animal model with the highest increase in publication [1]. This trend resulted from the importance of zebrafish as an economical, practical and a good scientific model for several human diseases [2]. It also has a huge potential for drug discovery and gene function identification through behavioural phenomics and high-throughput genetic and molecule screening [3].

The practicability, and scientific interest in zebrafish lies in the high genetic homology to humans (80–85%), reproductive success, embryos and larvae transparency, rapid development, and the lack of ethical restriction to the use of zebrafish before hatching [1]. Zebrafish is legally protected by the European Directive on the protection of animals used for scientific purposes (2010/63/EU) when they become capable of independent feeding [4], but even then, its use is considered a *relative replacement*, i.e. the use of an animal with a nervous system less complex than mammals [5].

However, this increasing use of zebrafish has not been accompanied by the evolution of proper anaesthesia for this species in research. Ethyl 3-aminobenzoate methanesulfonate (MS222) is the standard fish anaesthetic, widely used in zebrafish, but concerns have been raised regarding aversion and stress induction [6,7]. As a local anaesthetic, MS222 may act more as a muscular blocking agent rather than as an anaesthetic [8], and it may reduce heart rate and causes high mortality under long-term sedation [9]. Furthermore, MS222 needs special preparation and storage [10] which could make its use less practical.

Small fish as zebrafish are often anaesthetized in a water bath where the risk of poor anaesthesia or overdose is high. Indeed one of the challenges of zebrafish anaesthesia is the anaesthetic depth control. Hence more and better anaesthetic protocols are required to improve the use of zebrafish model in different experimental situations.

Propofol is a short-acting hypnotic agent, allowing a smooth anaesthesia induction and a quick recovery with little cumulative effects, but its efficacy and safety in small fishes as zebrafish is still poorly described [11,12]; while lidocaine is a sodium-channel blocker with large margin of safety in medaka [13], another small aquarium fish model.

Therefore, we aim to study the quality of anaesthesia and recovery induced by different concentrations of propofol alone and in combination with different concentrations of lidocaine, and to propose new anaesthetic protocols to be used in zebrafish anaesthesia. We predicted that the combination of propofol/lidocaine would confer a full anaesthesia (hypnosis and analgesia) to zebrafish without the need to adjust solution pH as MS222, increasing efficiency.

## Material and Methods

### Ethics statement

All procedures were carried out under personal and project licenses approved by the National Competent Authority for animal research, named Direção-Geral de Alimentação e Veterinária (DGAV, Lisbon, Portugal) (approval number: 017216), and by the Institutional Animal Care and Use Committee (IACUC) of IBMC and UTAD for a project where this study protocol were described. All experimental procedures were performed in accordance with the European Directive 2010/63/EU on the protection of animals used for scientific purposes, and its transposition to the Portuguese law, ‘Decreto Lei’ 113/2013.

### Animals and Housing

Ninety-five 1.5 years old AB zebrafish bred in the Animal Facility of the institute were used. They were maintained in a 20 L tank at 28 ± 0.5°C, pH = 7.3–7.5, in a 14:10 h light:dark cycle, and in a semi-closed water system with aeration and with mechanical and biological filtration. Fishes were fed twice a day with a commercial diet (Sera, Heinsberg, Germany), supplemented with artemia. After anaesthesia administration, the animals recovered individually for 24hours in a 5L tank (25x15x15cm) with water at 28 ± 0.5°C, and in visual contact with the neighbours. This system was a semi-closed water system with mechanical, biological, and carbon filters, with 100% water exchange per day and aeration. All tanks had UV sterilized water. The daily routine of the animal facility comprises the observation of general signs of health and welfare as the lack of food consumption, loss of equilibrium in the water, alteration of the mucosa colour, erratic movements for long periods of time, lack of swimming in the water column, unresponsiveness to touch or water agitation. During the experiment, the animals were monitored 1 hour, 5 hours, 24, and 48 hours after anaesthesia. The presence of erratic movements, and the lack of swimming in the water column were considered normal as prior anaesthesia treatment may have caused these signs.

### Experimental questions

**Experiment A: Can propofol alone or combined with lidocaine be an effective anaesthetic protocol for adult zebrafish?** Zebrafish were randomly assigned to 7 different groups: control (unanaesthetized animals, control A, n = 10); anaesthetized animals with low (2.5 μg/ml, 2.5P, n = 15), intermediate (5 μg/ml, 5P, n = 11), or high (7.5 μg/ml, 7.5P, n = 11) propofol (Lipuro 2%, B. Braun Melsungen AG, Germany) dose; and 2.5 μg/ml of propofol combined with low (50 μg/ml, P/50L, n = 10), intermediate (100 μg/ml, P/100L, n = 13) or high (150 μg/ml, P/150L, n = 11) lidocaine (1%, Braun, Queluz de Baixo, Barcarena, Portugal) dose. These anaesthetic solutions did not require any previous preparation.

After the evaluation of anaesthetic parameters and recovery, we targeted as a potentially good anaesthetic protocol the combination propofol/lidocaine. Thus, we had to compare this new protocol with the standard one, the anaesthetic MS222. Experiment B was then performed with this goal.

**Experiment B: Does the standard anaesthetic MS222 differ from a new anaesthetic protocol for adult zebrafish?** Adult zebrafish were randomly allocated into two groups: zebrafish treated with 100 mg/L of MS222 (group 100M, n = 8), and a control group of unanaesthetized animals (control B, n = 4). Buffered MS222 solution was prepared by adding ethyl 3-aminobenzoate methanesulfonate powder (Sigma-Aldrich, USA) to system water, making a stock solution of 10 g/L buffered with sodium bicarbonate until pH reached 7.0. Anaesthetic parameters of MS222 were evaluated by comparing them with the outcomes of the best anaesthetic protocols of experiment A, propofol/lidocaine combination. Activity of MS222-treated animals was compared with the control group B.

### Anaesthesia

In order to anaesthetize zebrafish, all the anaesthetic solutions previously referred were prepared in a beaker of 200 ml with an UV water sterilized at 28.2± 0.79°C, pH of ~8.2. After placing the corrected water in the beaker, the anaesthetics were placed in the middle of the water, and the solution was vigorously stirred; the drugs of the anaesthetic combination were placed individually. Zebrafish were then immediately placed in the prepared water bath and time to lose the equilibrium, the reflex to a mild touch and to a tail pinch were measured. Equilibrium lost was considered when fish stayed more than 3 seconds in dorsal recumbency. The response to a mild touch, a light stimulus, was evaluated by touching the lateral side of the fish with a pipette, and the response to a tail pinch was observed by gently pressing the caudal fin with forceps. Stimuli were tested every 10 seconds. After the loss of the tail pinch reflex or when 5 minutes elapsed from the loss of equilibrium, the animal was placed alone in a tank to recover. In half of the fishes of experiment A (n = 5), and in the fishes of experiment B, respiratory rate (RR) was measured 15 seconds after equilibrium loss, and 15 seconds after loss of response to a mild touch. Control animals were left in a 200 ml beaker with water without anaesthetics for ~1 minute to mimic the time spent by treatment groups until loss of equilibrium, and then they were placed in the same aquarium system as the other groups for further evaluation.

If some fish died during anaesthesia, the number would be increased to have at least 8–10 animals in each group to evaluate their recovery. Anaesthetic overdose is also an euthanasia method approved by the DGAV (national competent authority to evaluate the ethical use of animals in research).

### Post-anaesthetic recovery assessment

When the animal was placed in the tank to recover, the time until it starts moving and to recover the equilibrium were measured. Equilibrium recovery was defined as more than 3 seconds in ventral recumbency. Five and 24 hours post-anaesthesia (hpa), zebrafish were evaluated concerning activity in both experiments. Activity was measured by the frequency of crossing the longitudinal line of the tank per minute (crossings/min). Reactivity was assessed in experiment A by observing the response of the fish to the experimenter’s approach in a single movement, i.e. the experimenter advanced towards the tank in a rapid and single movement. Any reaction of the fish was recorded as “presence of response” (e.g.: increases agitation, moves away from or comes close to the experimenter, changes direction, freezes). After these evaluations, food was given and latency to eat was recorded in all points of time. For activity assessment, each experiment had a control group; P/L and P groups were compared with control of experiment A (control A) and 100M group was compared with control of experiment B (control B). Observations were done by a blind experimenter to the treatment of each fish. These assessments required individual housing for 24hours, but this isolation was minimized by keeping the fish in visual contact with other conspecifics. In the end, fish were euthanized using the concussion method followed by decapitation to ensure death, as approved by the ethics committee of DGAV for this experiment.

**Fig 1:**
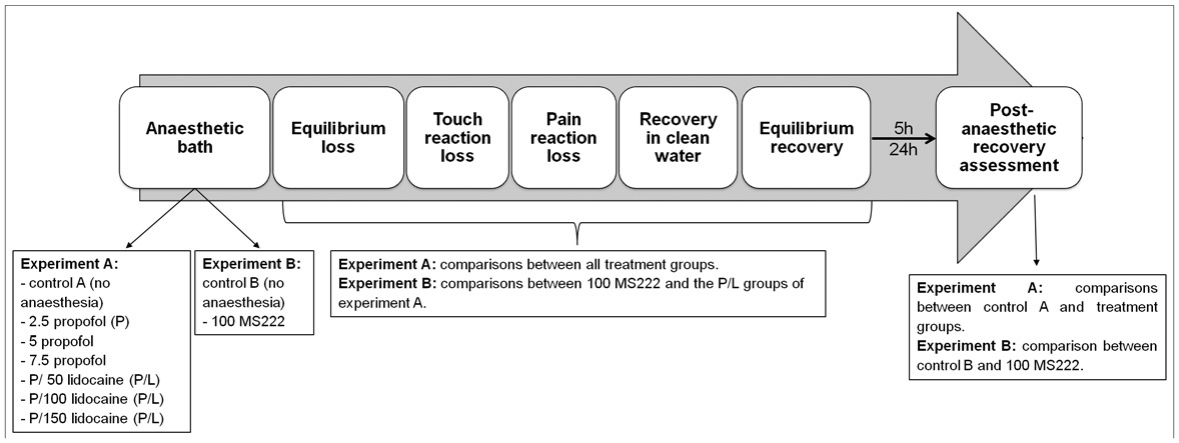
Scheme of the study workflow, groups used and the comparisons made between groups concerning the assessments performed. The post-anaesthetic recovery was assessed by the fish reactivity to the approach of the observer in experiment A, and by the fish activity (number of crossings per minute made in a virtual line) in both experiments.

A workflow of this experiment with a brief description of groups are presented in Fig 1.

### Statistics

**Experiment A**: Differences between groups regarding time to equilibrium loss and to recover, to lose the response to a light stimulus, to start moving after anaesthesia, respiratory rate, crossings/min (5hpa), and the presence or absence of reaction to observer approach were analyzed with Kruskal-Wallis and Dunn test to pairwise comparisons. One-way ANOVA with Tukey as a post-hoc test was used to evaluate time to lose response to the tail pinch, and crossings/min (24hpa). Likelihood ratio was used to test if there was an association between the number of animals to react to observer’s approach and the treatment groups.

**Experiment B**: Regarding anaesthetic parameters, the best protocols of experiment A (each P/L group) were compared with the standard anaesthetic MS222 (100M), using Mann-Whitney with Bonferroni corrections. Kruskal-Wallis and Dunn test to pairwise comparisons were used to study differences of respiratory rate between treatment groups (P/L and 100M groups). Crossings/min were analyzed using independent Student’s t-test for differences between control B and 100M group.

All hypotheses were two-tailed tested and statistical significance was considered to be reached at p≤ 0.05, except when Bonferroni corrections were applied (p< 0.01667). All results were analyzed by using Microsoft Office Excel 2007 (Microsoft Corporation, Redmond, WA, USA) for data acquisition, SPSS 20.0 for Windows (SPSS Inc., Chicago, IL, USA) for statistical analysis, and GraphPad Prism 6 for Windows (GraphPad Inc., San Diego, CA, USA) for graphical representations. Although there are parametric and non-parametric data, we chose to present all data as median [interquartile range].

## Results

All animals lost the equilibrium and recovered in water at similar temperatures.

### Experiment A

**Fig 2:**
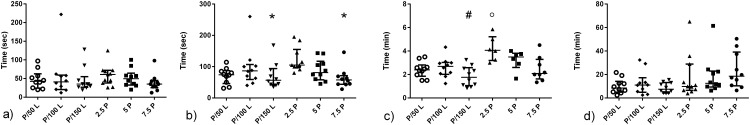
Anaesthetic parameters of adult zebrafish when treated with propofol alone or combined with lidocaine. a) equilibrium loss, b) loss of touch reaction, c) loss of reaction to a painful stimulus, and d) equilibrium recovery of adult zebrafish after being placed in an anaesthetic bath of 2.5, 5 or 7.5 μg/ml of propofol alone (P) or 2.5 μg/ml of propofol combined with 50, 100 or 150 μg/ml of lidocaine (P/L). Each point represents an animal. n = 10. Data are expressed as median [interquartile range]. * p< 0.05 compared with 2.5 μg/ml of propofol; ° p< 0.05 compared with all groups except with 5 μg/ml of propofol; ^#^p< 0.05 compared with 5 μg/ml of propofol.

No statistical differences were detected between treatment groups of propofol alone or combined with lidocaine regarding time to lose equilibrium, to start moving and to regain equilibrium. Only one fish from the lowest propofol dose (2.5 μg/ml—Group 2.5P) did not lose the light stimulus response. The other animals from group 2.5P took more time to lose response to a light stimulus than the ones from group 7.5P (p = 0.0066), and P/150L (p = 0.0278). Group 2.5P also took more time to lose response to a painful stimuli (tail pinch) than group 7.5P (p = 0.0092), P/50L (p = 0.004), P/100L (p = 0.0131) and P/150L (p< 0.0001). P/150L group was quicker to lose the tail pinch reflex than 5P group (p = 0.0473) (Fig 2).

The highest mortality was in the zebrafish group treated with the lowest propofol concentration wherein one fish died 48hours post-anaesthesia; no fish died in group P/50. Propofol alone was not sufficient to induce analgesia to all zebrafish. By the contrary, the lowest propofol dose combined with any of the lidocaine concentrations tested was sufficient to induce analgesia (Table 1).

**Table 1:** Proportion of animals that responded to the light and painful stimuli after 5 minutes of anaesthesia, and proportion of animals that died in each treatment group.

**Groups**	**Proportion fish responding to light stimulus**	**Proportion fish without analgesia**	**Proportion of mortality**
**2.5/50 μg/ml P/ L**	0/10	0/10	0/10
**2.5/100 μg/ml P/ L**	0/13	0/13	3/13
**2.5/150 μg/ml P/ L**	0/11	0/11	1/11
**2.5 μg/ml P**	1/15	6/15	5/15
**5 μg/ml P**	0/11	4/11	1/11
**7.5 μg/ml P**	0/11	2/11	1/11

P- propofol; L- lidocaine.

**Fig 3:**
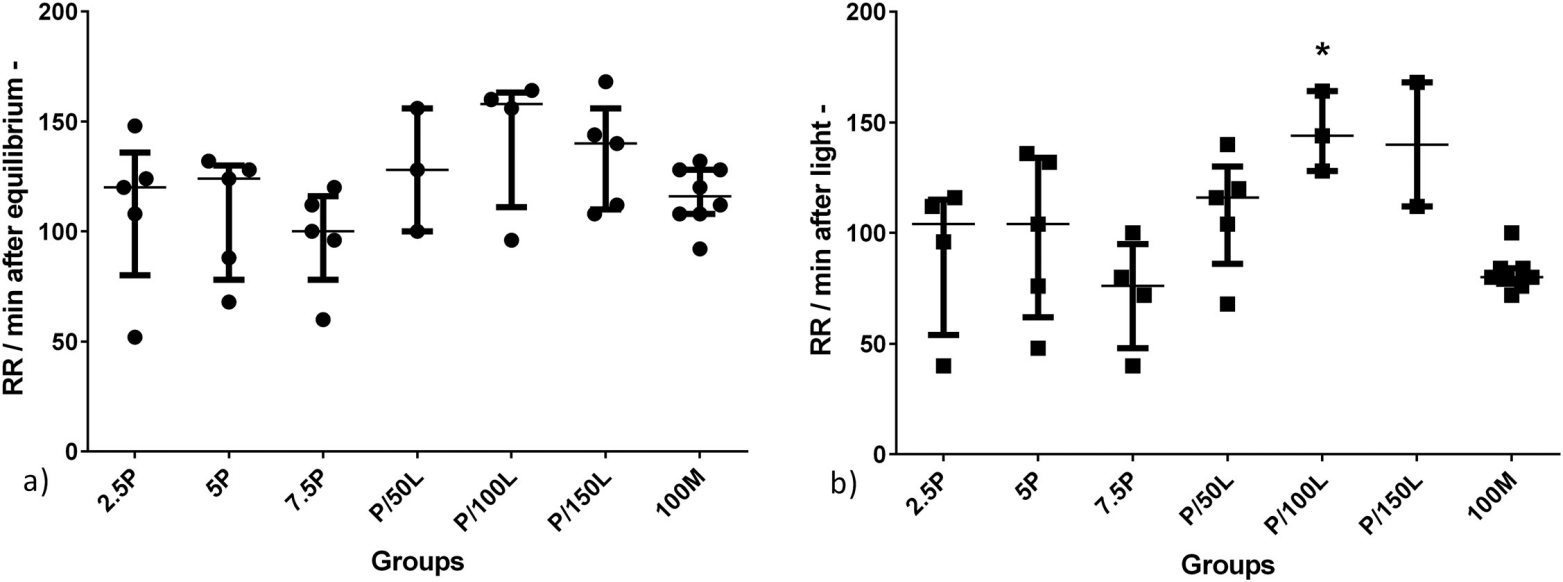
Respiratory rate per minute (RR) of adult zebrafish treated with the different anaesthetic protocols. a) RR after loss of equilibrium (equilibrium-), and b) RR after loss of light stimulus response (light-) of adult zebrafish treated with different concentrations (2.5, 5 or 7.5 μg/ml) of propofol alone (P) or 2.5 μg/ml of propofol combined with 50, 100 or 150μg/ml of lidocaine (P/L), or treated with 100 mg/L of MS222. Each point represents an animal; we were not able to count the respiratory movements of all animals. Data are presented as median [interquartile range].

No significant differences were found between groups (propofol alone or combined with lidocaine) regarding the respiratory rate per minute in any of the periods assessed. Nevertheless, after the loss of equilibrium and loss of the light stimulus reflex, there seems to be a dose dependent effect wherein the RR decreased with high propofol doses. The addition of lidocaine seemed to increase respiratory rate (Fig 3). In the Fig 3 not all the animals used (n = 5 for P and P/L groups) were represented, as the frequency of respiratory movements are not always easy to count by direct observation, especially when the rate is high as in P/L combinations.

**Fig 4:**
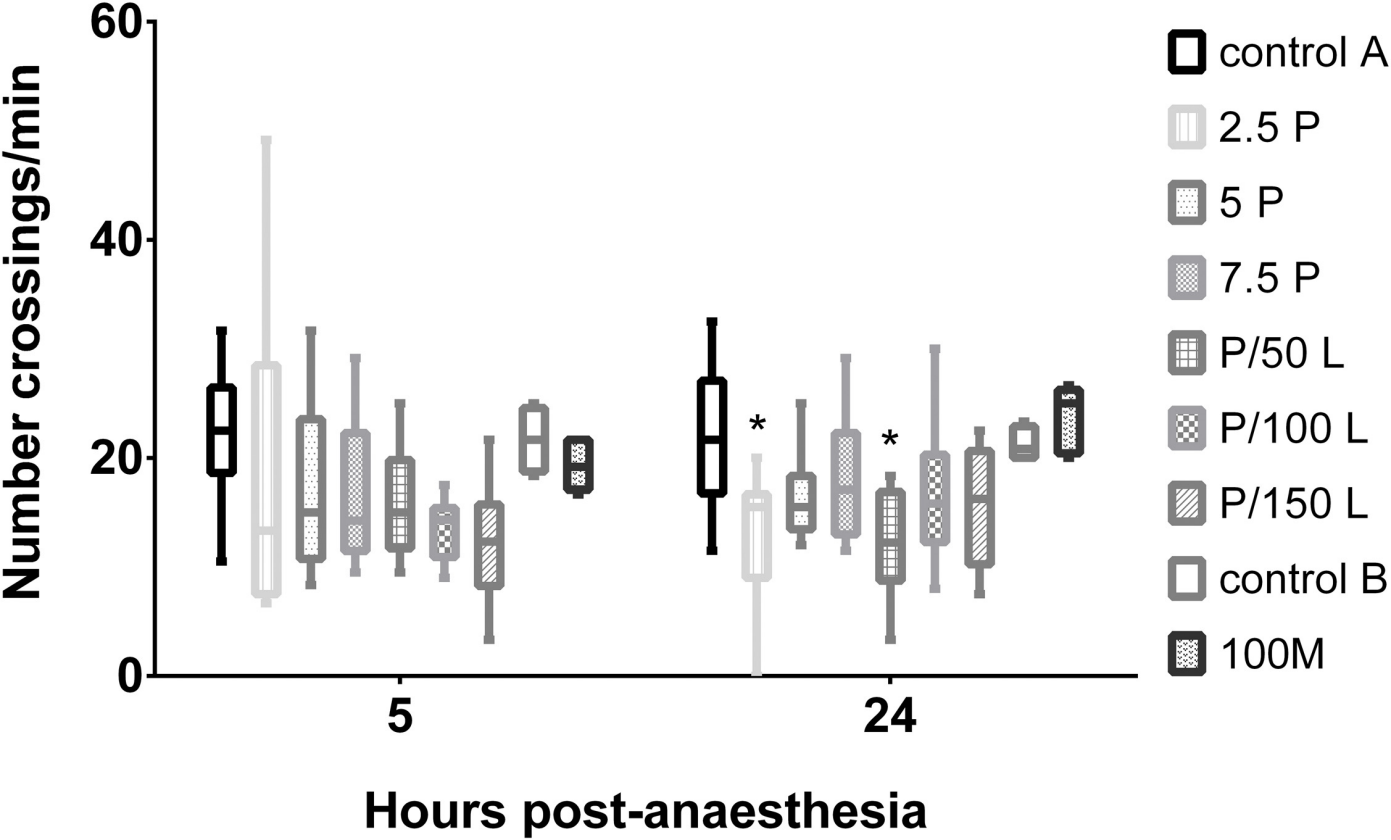
Number of crossings per minute of adult zebrafish through the middle/ longitudinal line of the tank 5 and 24 hours after being treated with the different anaesthetic protocols, and the control groups. Zebrafish were treated with 2.5, 5 or 7.5 μg/ml of propofol alone (P) (n = 10, except 2.5P with n = 11), 2.5 μg/ml of propofol combined with 50, 100 or 150 μg/ml of lidocaine (P/L) (n = 10), or treated with 100 mg/L of MS222 (100M, n = 8), and respective unanaesthetized controls (control A, n = 12; and control B, n = 4). Data are presented as a box plot (the median is indicated by the horizontal bar inside the box; the 25th and 75th percentile are the boxes’ borders; and the whiskers are the lowest and highest values for the 5th and 95^th^ percentiles, respectively). P and P/L groups were compared with control A, and 100M group was compared with control B. * p< 0.05 compared with control A.

Only 3 animals from 2.5P (one 1h post-anaesthesia and two 24h post-anaesthesia), and 1 animal from P/150L 1h post-anesthesia were at the bottom; all the other zebrafish swam in the water column. Zebrafish activity was assessed by the number of crossings through a virtual line 5 and 24 hours post-anaesthesia, as explained in the methodology. Five hours post-anaesthesia, no differences were detected between treatment groups and control group A. However, 24hpa, control A animals crossed more often the middle of the tank than P/50 (p = 0.005) and 2.5P group-treated animals (p = 0.003) (Fig 4). This last difference was statistically significant due to the immobility of two animals treated with 2.5 μg/ml propofol that also not reacted to the observer approach. The presence or absence of animals’ reaction to the observer approach was not associated with the treatment group, and there were no differences between groups regarding this measure (S1 Table); thus this assessment was not performed for experiment B.

Only propofol/lidocaine combinations induced full anaesthesia in all animals (loss of equilibrium and response to painful stimulus), therefore only P/L groups were compared with a standard dose of MS222 [6] in experiment B.

### Experiment B

All animals treated with MS222 lost all the reflexes tested and no fish died.

**Fig 5:**
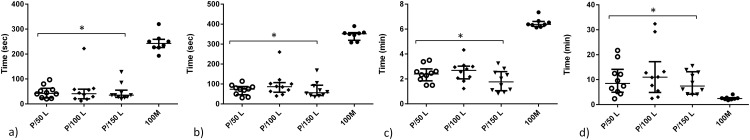
Anaesthetic parameters of adult zebrafish treated with propofol combined with lidocaine or treated with MS222. a) equilibrium loss, b) loss of touch reaction, c) loss of reaction to a painful stimulus, and d) equilibrium recovery of adult zebrafish after being placed in an anaesthetic bath of 2.5 μg/ml of propofol combined with 50, 100 or 150 μg/ml of lidocaine (P/L; n = 10), and of 100 mg/L of MS222 (100M; n = 8). Each point represents an animal. Data are expressed as median [interquartile range]. * p≤ 0.016 compared with 100 mg/L of MS222.

All the propofol/lidocaine concentrations induced a quicker loss of equilibrium, and loss of response to light and painful stimuli (p< 0.001) compared with MS222-treated fishes. On the other hand, MS222-treated fishes started moving (p≤ 0.016) and recovered the equilibrium (p≤ 0.002) faster than propofol/lidocaine groups (Fig 5).

There were no statistical differences regarding respiratory rate after equilibrium loss. After loss of response to a light stimulus, P/100L group had a significantly higher respiratory rate than 100M group (p = 0.029); no other differences were detected (Fig 3).

One hour, 5 hours and 24 hours after MS222 anaesthesia, all zebrafish swam in the water column. MS222-treated animals revealed similar activity to control B (Fig 4).

## Discussion

There are several limitations regarding drugs availability for anaesthesia in fish and zebrafish in particular. The most commonly used drug, MS222, has been recently described to induce aversion in zebrafish [6,7], and to reduce heart rate, increasing mortality [9]. These concerns were our motivation to test new protocols in zebrafish. In this study, we first intended to assess the clinical efficacy of propofol and propofol/lidocaine combination, a new approach in zebrafish (and other fish) anaesthesia. Subsequently, in a second phase of experiments, the anaesthetic parameters of the best protocol were compared with the animals anaesthetized with MS222.

In the study A, all zebrafish lost the equilibrium with the anaesthetic propofol or the combination propofol/lidocaine. Not all the concentrations of propofol used alone induced the loss of painful response, but when the lowest concentration of propofol was combined with lidocaine, analgesia was achieved in all cases. There were no differences between these protocols regarding time to recover. Zebrafish activity was fully normalized at 5 hours post-anaesthesia, and, sooner, 1h post-anaesthesia, all fishes had already swum in the water column and responded to food.

To our knowledge there are no studies using propofol in a water bath in zebrafish, nor using the combination propofol/ lidocaine. Using lidocaine alone may have the same limitation as using MS222, as both act as local anaesthetics. Thus, they may induce a neuromuscular blockage instead of a general anaesthesia, which we are not able to perceive. Nevertheless, lidocaine was already used in medaka with no side-effects [13], and it is often used as an analgesic, especially in large fishes as rainbow trout [14]. But its use in adult zebrafish induced a surgical plane of anaesthesia with a narrow safety margin. The only safe analgesic concentration of lidocaine took longer than MS222 to induce loss of equilibrium [15]. Therefore, the addition of a hypnotic, as propofol, to lidocaine seems to be an effective procedure to potentiate loss of equilibrium.

Propofol is a short-acting, intravenously administered hypnotic agent, rapidly metabolized and less prone to cumulative effects [11]. Its use includes the induction and maintenance of general anaesthesia, sedation for mechanically ventilated adults, and procedural sedation. Propofol is generally considered a very safe drug in other vertebrates as mammals and is commonly used in veterinary medicine. However, propofol is not commonly used in fish anaesthesia but there are some reports of its intravenously application in large fishes [16], and in a water bath [11,17,18], inducing a light plane of anaesthesia with a short induction time. Propofol also seems promising for fish sedation before transport, as it prevented the peak of cortisol levels, and preserved the hematological, morphological, and biochemical stability [19,20]. The consensus regarding propofol effects observed between species may be explained by the gene conservation of some hypnotic pathways between mammals and zebrafish, as the one using GABBA A receptors, the pharmacological binding site of propofol [21]. The propofol kinetic properties, namely its rapid metabolization, may be extremely useful in the case of zebrafish anaesthesia to ease anaesthetic depth control. Fish are usually fully immersed in the anaesthetic solution that is absorbed through the gills and skin being difficult to prevent overdose. Rapid propofol metabolization was already showed in rainbow trout, wherein propofol absorption and elimination were high, with a half-life time of 1.1 h at 17°C, a value that may decrease in water at higher temperatures [17].

In this study, different concentrations of propofol, and lidocaine combined with propofol did not cause different induction times of anaesthesia nor different times to recover. The difference was related to analgesia achievement that was induced in all fishes and quickly achieved with the addition of lidocaine to propofol or when propofol concentration was increased. After the loss of equilibrium or after the loss of light stimulus reflex, the respiratory rate decreased with high propofol doses, and increased when lidocaine was added to propofol. This effect was expected as propofol may induce respiratory depression [16]. Therefore, to achieve analgesia, it is advisable to use propofol combined with lidocaine, allowing to decrease the propofol dose for a safer analgesia and anaesthesia. This approach is called balanced anaesthesia wherein two drugs potentiate to produce the desired effects, while reducing the risk of side-effects, such as hemodynamic instability and mortality [22]. Also, lidocaine decreases the pH of the solution containing propofol [23]. This lower pH may increase the percentage of non-ionized drug in solution, inducing a faster onset of action [24]. Thus, these drugs may induce a synergetic effect, resulting in a rapid full anaesthesia.

Indeed, the combination propofol/lidocaine induced a quicker loss of equilibrium, and loss of response to light and painful stimuli compared with a standard dose of MS222 in adult zebrafish. On the other hand, MS222 induced a quicker recovery/ equilibrium gain compared with propofol/lidocaine protocol. This was expected, as the induction of a lighter anaesthetic depth facilitates the response of the body to return to its normal state of consciousness. The described stimulatory effect of MS222 on the cardiac and respiratory system of fish [25] were only observed after the equilibrium loss. Later on, after the loss of response to light stimulus, zebrafish treated with MS222 had a lower respiratory rate compared with the ones treated with the high doses of propofol/lidocaine. In fact, MS222 was also described to depress cardiovascular and respiratory function due to the blockage of ion channels in several cells, depending on the anaesthesia duration [26].

Concerning activity, the animals recovered quickly; all groups responded similarly to the control group when the external stimuli, observer approach and food, were applied 1 hour after anaesthesia. This indicates a quick behavioural recovery after using these anaesthetic protocols. The activity of MS222-treated animals was at control levels in all points of time. However, 24hpa, 2.5P group had two animals that not moved nor responded to external stimuli, which may be a long-term sedative effect of propofol at low concentrations. At 24hpa, P/50L group had less activity than the control A, but all the animals swam in the water column and reacted to external stimuli; in this case a more accurate analysis, as video recording, would be needed.

There seemed to be a high variability within propofol groups as regards time until equilibrium recovery. The addition of lidocaine to propofol reduced this variability and a more consistent result of individuals within P/L groups were observed. Propofol is delivered in a lipid emulsion rapidly distributed into peripheral tissues [27], and the drug washout for zebrafish recovery may depend on the body mass and lipid quantity present in each animal. Moreover, as an emulsion, propofol may not be equally distributed in the water and the solubilization may differ, despite the agitation that was performed. The addition of lidocaine may have altered the propofol conformation in a way that the previously referred limitations were minimized and the individual animal response to anaesthesia were more standardized. Some of the alterations described are the pH decrease from 7.5 with propofol to 6 with lidocaine [28], and the formation of enlarge propofol droplets [29]. However studies showed that propofol/lidocaine may be safely used within 30 minutes of preparation with no clinically important alterations in anaesthetic and sedative efficacy of propofol [30]; the droplets in emulsion only increased significantly after 30 minutes to 1 hour of lidocaine addition [31]. We always used a freshly prepared solution and the zebrafish stayed there for a maximum of 5 minutes. In our study, lidocaine seemed to stabilize propofol in solution, obtaining more consistent responses between different animals.

During experiments, fishes are often handled and subjected to treatments during which they are removed from their element, water, and transiently unable to breath. Therefore, the use of propofol alone, even not always inducing analgesia, may be especially useful in this animal model to increase animal welfare by inducing sedation during stressful events for fish.

Our aim to propose a new anaesthetic protocol inducing a quick and full anaesthesia was achieved by the propofol/lidocaine combination, which induced anaesthesia in less than 3 minutes, a consistent response within animals as regards to anaesthesia recovery, and normal activity 5 hours post-anaesthesia. M222 also fulfilled the referred requirements to an anaesthetic protocol. From the user perspective, all these drugs are easily available for research but propofol/lidocaine had lower costs and is easier to prepare compared to MS222. In powder, MS222 requires to be weighed (a fume hood is advisable), and a stock solution prepared. This solution needs to be buffered to neutralize MS222 acidity [10]. Moreover, there are also some storage requirements, and the loss of potency throughout time is still not consensual among researchers and vendors [32].

In this study we just used one strain and middle aged zebrafish, but other strains and stages of life of zebrafish may respond differently to these anaesthetic protocols. Thus these protocols should always be tested in a small number of animals from a specific strain and age prior to the experiment in order to ensure the anaesthetics’ clinical efficacy and safety.

In conclusion, the standard MS222 and the new protocol propofol/lidocaine showed advantages in different features. Propofol/lidocaine showed a potential to be used when we need a rapid loss of equilibrium to perform a painful procedure, for example, tail fin collection. This anaesthetic protocol does not need any special preparation. MS222 provide a quick recovery (equilibrium gain and activity), which is practical and ideal when there is a need to observe the immediate effect of a procedure, for example, intraperitoneal injection of a certain substance. Thus, these results showed the importance to optimize different anaesthetic protocols adequate for different experimental situations.

## Supporting Information

S1 Table Proportion of animals that responded to the stimulus observer approach 1, 5 and 24 hours post-anaesthesia. (PDF)
