# Supplementary material for: A New Anaesthetic Protocol for Adult Zebrafish (Danio rerio): Propofol Combined with Lidocaine
Source: PLoS One. 2016 Jan 25;11(1):e0147747. doi: 10.1371/journal.pone.0147747 (PMC4725851; doi:10.1371/journal.pone.0147747)
Supplement: S1 Table — (PDF) [file pone.0147747.s001.pdf]

Supporting information

S1Table: Proportion of animals that responded to the stimulus observer approach 1, 5 and 24 hours post-anaesthesia (hpa). P- propofol; L- lidocaine.

Groups	Proportion fish responding to observer approach		
	**1hpa**	**5hpa**	**24hpa**
Control	10/12	10/12	10/12
2.5/50µg/ml P/ L	5/10	9/10	7/10
2.5/100µg/ml P/ L	5/10	9/10	9/10
2.5/150µg/ml P/ L	4/10	8/10	9/10
2.5µg/ml P	6/11	9/11	9/11
5µg/ml P	8/10	8/10	8/10
7.5µg/ml P	5/10	9/10	8/10
